# Percutaneous medial hemi-epiphysiodesis using a transphyseal screw for caput valgum associated with developmental dysplasia of the hip

**DOI:** 10.1186/s12891-017-1833-5

**Published:** 2017-11-14

**Authors:** Chang Ho Shin, Wan Kee Hong, Doo Jae Lee, Won Joon Yoo, In Ho Choi, Tae-Joon Cho

**Affiliations:** 1 Department of Orthopaedic Surgery, Seoul National University College of Medicine, Seoul, Republic of Korea; 2 Division of Pediatric Orthopaedics, Seoul National University Children’s Hospital, 101 Daehak-ro Jongno-gu, Seoul, 03080 Republic of Korea

**Keywords:** Developmental dysplasia of the hip, Caput valgum, Hemi-epiphysiodesis, Transphyseal screw

## Abstract

**Background:**

The purpose of this study was to evaluate the radiologic outcome of percutaneous medial hemi-epiphysiodesis using a transphyseal screw for the management of caput valgum associated with developmental dysplasia of the hip (DDH).

**Methods:**

Eighteen hips (18 patients) having caput valgum treated with screw hemi-epiphysiodesis were followed for more than 2 years, and were included in this study. The mean age at the time of the index operation was 8.3 years (range, 4.3 to 10.7 years) and age at the latest follow-up was 12.2 years (range, 9.4 to 16.4 years). The screw in 5 hips was changed into a longer one at postoperative 21.8 months (range, 14 to 29 months) because the proximal femur outgrew the screw. The screws in 11 hips were removed at the mean age of 10.9 years (range, 8.0 to 14.5 years). We retrospectively analyzed the change in various radiologic parameters over time.

**Results:**

The mean Hilgenreiner-epiphyseal angle (HEA) of the operated side was 5.1 ± 11.3° preoperatively, and increased to 20.6 ± 11.3° at the latest follow-up (*p* = 0.001). The mean difference of the HEA between the operated and contralateral sides was 16.9 ± 15.1° preoperatively, which decreased to 2.4 ± 12.4° at the latest follow-up (*p* = 0.008). The mean articulo-trochanteric distance of the operated side, which was 3.2 ± 5.5 mm longer than that of the contralateral side preoperatively, became 5.6 ± 9.1 mm shorter at the latest follow-up (*p* = 0.001). The ratio of femoral neck length of the operated side to that of the contralateral side decreased over the follow-up period. Acetabular shape as measured by the Sharp angle and acetabular roof angle and femoral head coverage as measured by lateral center-edge angle did not change significantly by the index operation. The ratio of medial joint space width of the operated side to that of the contralateral side did not change significantly.

**Conclusions:**

Screw medial hemi-epiphysiodesis can effectively correct caput valgum associated with DDH. However, this technique remains coxa brevis and does not seem to significantly affect acetabular morphology or reduce subluxation.

## Background

Osteonecrosis of the capital femoral epiphysis, resulting in growth disturbance of the proximal femur, is a common and major complication secondary to treatment of developmental dysplasia of the hip (DDH) [1–5]. Of four types of growth disturbance classified by Kalamchi and MacEwen, type II or lateral growth disturbance, which causes valgus tilt appearance of the femoral head on the neck, is the most common [6]. As a result of continued normal medial growth, the femoral head and longitudinal growth plate tilt laterally [7].

Although Kim et al. reported that lateral growth disturbance was not necessarily associated with poor acetabular remodeling [8], caput valgum could induce compromised acetabular index, increase uncovered portion of the femoral head, and promote subsequent acetabular labral tears and early-onset osteoarthritis [8–11]. In addition, it positions the fovea capitis femoris, with no hyaline cartilage, more superior and lateral to its original position, that was previously positioned slightly posterior and inferior to the center of the articular surface [12]. In the severe case of caput valgum, the femoral head could change to “cocked hat” deformity and be laterally subluxated [7].

Proximal femoral varus osteotomy (PFVO) could be a treatment option to correct caput valgum deformity caused by lateral growth disturbance. However, because PFVO is usually performed at the level of the lesser trochanter, away from the center of rotation of angulation, it occasionally requires a great amount of varization to correct deformity and leads to greater trochanter overriding. In addition, it is sometimes difficult to decide on the amount of varus when performing PFVO because it is hard to predict whether further deformity develops until skeletal maturity [13].

Guided growth of the proximal femur using a transphyseal screw across the inferomedial aspect of the proximal femoral epiphyseal plate is an alternative treatment option for caput valgum. Although guided growth using a transphyseal screw has been widely used after introduced by Métaizeau in 1998, a few animal studies reported the effect of medial hemi-epiphysiodesis using a transphyseal screw in skeletally immature hips [14–16]. To the best of our knowledge, there have been only two studies involving screw medial hemi-epiphysiodesis for lateral growth disturbance of the proximal femur followed by treatment of DDH [13, 17]. One of these studies mainly focused on a technique of deformity measurement and not on treatment outcome [17].

The purpose of this study was to evaluate the radiologic outcome of percutaneous medial hemi-epiphysiodesis using a transphyseal screw for caput valgum associated with DDH.

## Methods

We collected cases of proximal femoral lateral growth disturbance associated with DDH presenting with progressive caput valgum that were treated with percutaneous medial hemi-epiphysiodesis using a transphyseal screw. Between August 2009 and December 2014, they were treated in a tertiary-care children’s hospital and followed for more than 2 years. Hips associated with neuromuscular disease, skeletal dysplasia or congenital anomaly of other organs/systems were excluded. There was no teratologic hip dislocation or hip dislocation combined with arthrogryposis. Based on these criteria, 18 hips (18 patients) became the subjects of this study and medical records and serial radiographs were reviewed and analyzed.

There were 14 female (78%) and 4 male (22%) patients. Fourteen hips (78%) were unilateral DDH, and three hips (17%) were bilateral DDH with only one hip developing caput valgum. The remaining patient had bilateral DDH, whose both hips developed caput valgum and were treated with screw medial hemi-epiphysiodesis. One side was randomly selected and included in the study. Of 14 unilateral cases, 9 (64%) were right hips and 5 (36%) were left hips. The mean age (± standard deviation) was 8.3 ± 1.9 years (range, 4.3 to 10.7) at the time of screw placement, and 12.2 ± 2.0 years (9.4 to 16.4) at the time of the latest follow-up. The duration of follow-up averaged 3.9 ± 1.4 years (2.0 to 6.9).

Surgical procedures which were performed before, concurrent with, or after screw medial hemi-epiphysiodesis, are listed in Table 1. No proximal femoral osteotomy or pelvic osteotomy, that can alter acetabular or femoral morphology, was performed after hemi-epiphysiodesis. Applied surgical technique was similar to Lee et al.’s method [18]. A screw was placed through inferomedial one third to one fourth of the proximal femoral physis on the anteroposterior (AP) view and through the center of the physis on lateral view. The number of the threads of the screw that were placed across the physis was more than three, and we stopped advancing the screw when the tip of the screw reached the subchondral bone. After inserting a screw, intraoperative arthrogram was routinely performed to confirm the position of the screw tip.

**Table 1:** Treatment history of the hips

	*N* = 18^a^
Reduction method	
Closed reduction under general anesthesia	2 (11%)
Open reduction	16 (89%)
Procedures performed before hemi-epiphysiodesis	
Femoral varus osteotomy	3 (17%)
Salter innominate osteotomy	1 (6%)
Dega osteotomy	5 (28%)
Femoral varus osteotomy and Dega osteotomy^b^	5 (28%)
Femoral varus osteotomy and shelf aectabuloplasty^c^	1 (6%)
Femoral varus osteotomy, Dega osteotomy, and triple innominate osteotomy^c^	1 (6%)
Procedures concurrent with hemi-epiphysiodesis	
Dega osteotomy	1 (6%)
Shelf acetabuloplasty	1 (6%)
Epiphysiodesis, distal femur	2 (11%)
Procedures performed after hemi-epiphysiodesis	
Epiphysiodesis, distal femur	1 (6%)
Greater trochanter apophyisodesis	1 (6%)

^a^The values are given as the number of hips

^b^Procedures were performed at the same time in 4 hips and in sequence in 1 hip

^c^In sequence

Anteroposterior radiographs of the pelvis were taken with the hip positioned in neutral rotation and neutral abduction/adduction at each visit. We measured indicators of pelvic alignment in all radiographs. The quotient of pelvic rotation indicating pelvis position in the horizontal plane was 1.01 ± 0.18 (0.68 to 1.46) and the symphysis os-ischium angle, indicating the pelvis position in the sagittal plane, was 99.9° ± 8.7° (90.0° to 115.1°), which was considered acceptable [19, 20].

**Fig. 1:**
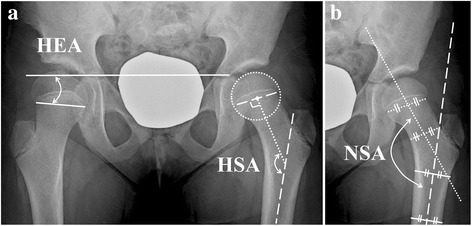
**a** Hilgenreiner-epiphyseal angle is the angle between the Hilgenreiner line and a line connecting the medial and lateral end of the proximal femoral physis on the hip anteroposterior radiograph. Head-shaft angle is the angle between the proximal femoral shaft axis and a line which is drawn though the center of the proximal femoral epiphysis and perpendicular to the proximal femoral growth plate. Hilgenreiner-epiphyseal angle of the right hip is 6 degrees, and head-shaft angle of the left hip is 152 degrees on this radiograph. **b** Neck-shaft angle is the angle between the axis of the proximal femoral shaft and the axis of the femoral neck, which links the midpoints of neck diameter at both levels of the subcapital and the base of neck areas. Neck-shaft angle of the left hip is 140.5 degrees in this radiograph

For evaluating proximal femoral morphology, the Hilgenreiner epiphyseal angle (HEA) [21], head-shaft angle (HSA) [22], and neck-shaft angle (NSA) [14] were measured (Fig. 1). For evaluating femoral neck length, the articulo-trochanteric distance (ATD) [23] and femoral neck length ratio [24] was measured and calculated, respectively. For evaluating acetabular morphology and subluxation, Sharp angle [25], acetabular roof angle [26], lateral center-edge angle (LCEA) [27], medial joint space (MJS) width ratio [28], and center-head distance discrepancy (CHDD) [29] were measured. Femoral neck length ratio and MJS width ratio were calculated by dividing the value of the operated side by the value of the contralateral side. Femoral neck length ratio and CHDD were measured only in the unilaterally affected 14 hips. The difference of radiologic parameters between the operated and contralateral sides in unilaterally affected hips were also calculated. Changes in the parameters from time of screw placement to the time of latest follow-up, as well as changes of the differences of those values between both hips, were obtained. Two hips, which underwent pelvic osteotomy concurrent with hemi-epiphysiodesis, were excluded from the analysis reflecting changes in parameters in acetabular morphology and subluxation. Skeletal maturity at the time of screw removal was determined based on the closure of the proximal femoral growth plate and triradiate cartilage.

Leg-length-discrepancy (LLD) was evaluated by iliac crest height difference measured on standing AP radiograph of the pelvis. Four patients with bilateral DDH and two patients who underwent epiphysiodesis of the distal femur concurrent with screw medial hemi-epiphysiodesis of the proximal femur were excluded from the analysis of LLD. The patient who underwent epiphysiodesis of the distal femur at the latest follow-up was included in the analysis.

To determine intra-observer reliability, measurements were made by one of the authors (W.K.H.) on two different days, 2 weeks apart. To determine the inter-observer reliability, the same measurements were made by another author (C.H.S.) after a consensus building session to define the radiographic measurements. Intra-observer and inter-observer reliability were evaluated by intraclass correlation coefficients (ICCs), which were calculated assuming absolute agreement and a single measurement with a 2-way-random-effects-model. In the intra-observer reliability test, ICCs were 0.988 (95% confidence interval [CI], 0.967, 0.995) for HEA, 0.957 (0.890, 0.984) for HSA, 0.917 (0.796, 0.968) for NSA, 0.909 (0.766, 0.965) for Sharp angle, 0.973 (0.946, 0.986) for acetabular roof angle, 0.780 (0.511, 0.911) for LCEA, and 0.961 (0.919, 0.982) for MJS width ratio. In the inter-observer reliability test, the ICCs for the same parameters were 0.871 (0.694, 0.950), 0.830 (0.607, 0.932), 0.903 (0.759, 0.963), 0.919 (0.799, 0.969), 0.826 (0.597, 0.931), 0.715 (0.510, 0.844), 0.762 (0.466, 0.904), and 0.886 (0.769, 0.945), respectively.

The Mann-Whitney U test was used to evaluate the significance of the difference between mean values between operated and contralateral sides. The Wilcoxon signed-rank test was used to evaluate the significance of change of parameters over time. *P* values <0.05 were considered significant.

## Results

The changes of radiologic parameters between operated and contralateral hips from the index operation to the latest follow-up are presented in Tables 2 and 3. In the proximal femoral morphologic domain, mean HEA of the operated hips increased significantly since the index operation (*p* = 0.001). The change of HEA ranged from −6.1° to 34.6°. However, that of the contralateral hips did not change significantly. The difference of HEA between the operated and contralateral sides decreased significantly (*p* = 0.008) (Table 4), of which the respective average became close to zero degrees at latest follow-up.

**Table 2:** Preoperative and postoperative data of the operated hips

*N* = 18	Preoperative status^a^	Latest follow-up^a^	*P* value†
Proximal femur			
Hilgenreiner-epiphyseal angle (°)	5.1 ± 11.3	20.6 ± 11.3	0.001
Head-shaft angle (°)	169.5 ± 10.5	150.0 ± 10.0	<0.001
Neck-shaft angle (°)	143.0 ± 7.9	132.1 ± 6.0	<0.001
Femoral neck			
Articulo-trochanteric distance (mm)	21.1 ± 7.9	13.6 ± 8.1	0.001
Femoral neck length ratio^b^	0.929 ± 0.173	0.787 ± 0.281	0.056
Acetabular morphology & subluxation^c^			
Sharp angle (°)	46.7 ± 4.8	44.9 ± 5.5	0.030
Acetabular roof angle (°)	17.1 ± 7.4	16.3 ± 8.0	0.408
LCEA (°)	23.3 ± 6.1	23.9 ± 8.0	0.717
Medial joint space width ratio^b^	1.408 ± 0.518	1.396 ± 0.489	0.507
CHDD^b^ (%)	9.2 ± 5.5	6.2 ± 5.1	0.003

†Wilcoxon signed rank test

^a^The values are given as the mean and the standard deviation

^b^Unilateral cases only (*N* = 14)

^c^Two hips which underwent pelvic osteotomy concurrent with hemi-epiphysiodesis were excluded

**Table 3:** Preoperative and postoperative data of the contralateral hips

*N* = 14	Preoperative status^a^	Latest follow-up^a^	*P* value†
Proximal femur			
Hilgenreiner-epiphyseal angle (°)	22.4 ± 5.9	23.0 ± 5.4	0.802
Head-shaft angle (°)	154.3 ± 5.4	152.7 ± 6.9	0.221
Neck-shaft angle (°)	138.8 ± 6.3	133.0 ± 5.3	0.005
Femoral neck			
Articulo-trochanteric distance (mm)	18.4 ± 3.4	18.9 ± 4.8	0.777
Femoral neck length ratio	Not applicable	Not applicable	
Acetabular morphology & subluxation			
Sharp angle (°)	48.7 ± 3.8	44.7 ± 5.5	0.002
Acetabular roof angle (°)	12.6 ± 4.9	7.4 ± 7.3	0.002
LCEA (°)	24.7 ± 5.6	31.6 ± 5.7	0.002
Medial joint space width ratio	Not applicable	Not applicable	
CHDD (%)	Not applicable	Not applicable	

†Wilcoxon signed rank test

^a^The values are given as the mean and the standard deviation

**Table 4:** Differences of radiologic parameters between the operated and contralateral sides

*N* = 14	Preoperative status^a^	Latest follow-up^a^	*P* value†
Proximal femur			
Difference of HEA^b^ (°)	16.9 ± 15.1	2.4 ± 12.4	0.008
Difference of HSA^c^ (°)	15.5 ± 11.2	−2.5 ± 13.0	0.001
Difference of NSA^c^ (°)	5.5 ± 5.6	0.9 ± 6.6	0.056
Femoral neck			
Difference of ATD^c^ (mm)	3.2 ± 5.5	−5.6 ± 9.1	0.001
Difference of femoral neck length ratio	Not applicable	Not applicable	
Acetabular morphology & subluxation^d^			
Difference of Sharp angle^c^ (°)	−1.9 ± 6.2	0.33 ± 7.7	0.187
Difference of acetabular roof angle^c^ (°)	3.3 ± 6.1	8.3 ± 9.3	0.011
Difference of LCEA^b^ (°)	2.1 ± 7.8	8.1 ± 9.1	0.009
Difference of CHDD (%)	Not applicable	Not applicable	

†Wilcoxon signed rank test

^a^The values are given as the mean and the standard deviation

^b^These values were calculated by subtracting the values of the operated hips from the values of the contralateral hips

^c^These values were calculated by subtracting the values of the contralateral hips from the values of the operated hips

^d^Two hips which underwent pelvic osteotomy concurrent with hemi-epiphysiodesis were excluded

Preoperatively, the operated side had significantly larger HSA than the contralateral side (*p* < 0.001), while the NSA was not significantly different between the sides (*p* = 0.085). The HSA of the operated side significantly decreased after the index operation but that of the contralateral side did not (*p* < 0.001 and *p* = 0.221, respectively). The NSA significantly decreased on both sides after the index operation, but the amount of change was not significantly different between the two sides (*p* = 0.285). At the latest follow-up, the means of both HSA and NSA became close to zero degrees.

The mean ATD of the operated side was 3.2 ± 5.5 mm longer than that of the contralateral side preoperatively which significantly decreased during follow-up (*p* = 0.001). Meanwhile, the ATD of the contralateral side changed little. As a result, the mean ATD of the operated side was 5.6 ± 9.1 mm shorter than that of the contralateral side at the latest follow-up. Femoral neck length ratio also decreased in 11 hips (79%) (*p* = 0.056).

Regarding acetabular morphology and subluxation, the Sharp angle significantly decreased on both sides, but the amount of change between them was not significantly different (*p* = 0.401) (Tables 2 and 3). Over the follow-up period, Acetabular roof angle and LCEA improved significantly on the contralateral side from normal growth of the acetabulum, while those of the operated side did not show significant changes. MJS width ratio did not change significantly. The mean CHDD improved after the index operation but remained over 6% at the latest follow-up (Table 2).

The screws were changed into longer ones, in five of the hips during the follow-up period postoperatively at 21.8 ± 4.7 months (range, 14 to 29 months), because the proximal femora outgrew the screws. Patient’s age at the index operation was 6.1 ± 1.6 years (4.3 to 7.7). The HEA increased until the revision surgery by 13.1° ± 5.7° (4.9° to 19.1°) and increased until the latest follow-up by 10.1° ± 5.8° (2.8° to 16.6°) over the mean period of 34 ± 25 months (2 to 60).

Screws were removed in 11 of 18 hips at the mean age of 10.9 ± 2.0 years (8.0 to 14.5). Six of the 11 hips underwent removal of the screw before skeletal maturity at the mean age of 9.6 ± 1.4 years (8.0 to 11.3) and were followed for more than 6 months after screw removal. In these six hips, HEA and HSA did not significantly change from the time of screw removal to the latest follow-up over the mean period of 24.5 ± 12.4 months (6 to 42).

The mean length of the operated legs was 12.6 ± 6.2 mm (0 to 22) longer than that of the contralateral legs preoperatively and 3.2 ± 8.8 mm (−11.6 to 19.7) longer at the latest follow-up. The mean growth of the operated legs significantly diminished compared to that of the contralateral legs over the follow-up period (*p* = 0.006).

There were no complications associated with screw hemi-epiphysiodesis such as penetration of the articular surface by the screw, chondrolysis, proximal femoral fracture, irritation symptom at the screw insertion site, or infection.

## Discussion

In this study, we reported the outcome of percutaneous medial hemi-epiphysiodesis using a transphyseal screw to correct caput valgum associated with DDH. We quantified caput valgum deformity using HEA and HSA, which were significantly different between the operated and contralateral sides before the index operation. These parameters on the operated hips became similar to those on the contralateral normal hips at the latest follow-up. We tried to evaluate the change of ‘fovea valga’ over the follow-up period, but could not complete this evaluation because the fovea capitis could not be delineated in many cases; probably due to the altered anatomy of the hip with type 2 osteonecrosis. Our data concur with those of Torode et al., which reported increase of proximal femoral physeal orientation and HSA [13] and McGillion et al. showed improvement of the HEA [17]. Along with the previous studies, taken collectively, our data support the efficacy of screw hemi-epiphysiodesis for caput valgum associated with DDH although previous studies and out data could not prove that this intervention improves the long-term prognosis regarding prevention of osteoarthritis.

In our study, preoperative HSA on the operated side was significantly larger than that of the contralateral side, but NSA was not. This means that the caput valgum deformity was not necessarily associated with coxa valga. After screw hemi-epiphysiodesis, HSA of the operated side decreased much more than that of the contralateral side. However, the decrease of NSA was not significantly different between sides. This result was not concordant to previous animal studies reporting a significant decrease of NSA by screw medial hemi-epiphysiodesis [14–16]. This discrepancy might arise from anatomical differences between animals and humans, or from differences in the measurement methods for NSA. There are several different definitions of NSA [30] and in our study, NSA was measured based upon femur neck orientation [14]. Our data suggest that screw medial hemi-epiphysiodesis corrects mainly caput valgum rather than coxa valga.

In the present study, the ATD of the operated side was longer than that of the contralateral side preoperatively as a result of caput valgum deformity, which became shorter than that of the contralateral side at the latest follow-up. Moreover, femoral neck length ratio also decreased over the follow-up period. This finding concurs with that of the animal study by McCarthy et al. [16] and implies that screw medial hemi-epiphysiodesis cannot prevent shortening of the femoral neck and that it remains a coxa brevis deformity even after the correction of the caput valgum.

In our study, the changes of the Sharp angle on the operated and contralateral sides were similar regardless of screw placement, which was close to the natural course of unoperated cases of caput valgum in a previous study [9]. Furthermore, acetabular roof angle and LCEA did not exhibit a significant improvement on the operated side although it is reported to improve along with normal growth of acetabulum [31, 32]. MJS width ratio also changed very little, and the CHDD remained over 6% in predicting poor acetabular development even after the index operation [29]. Based on these results, screw medial hemi-epiphysiodesis did not make significant impacts on acetabular morphology and femoral head coverage. Lack of significant differences might be because screw placement was performed at relatively older ages when only limited acetabular remodeling potential remained [32].

**Fig. 2:**
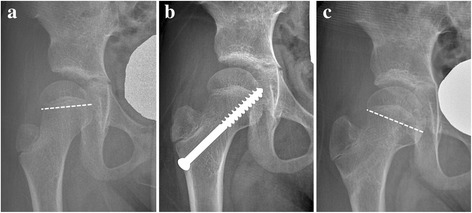
**a** A 8.2-year-old female patient was treated for developmental dysplasia of the hip by open reduction at age 2.0 years, showing caput valgum with Hilgenreiner-epiphyseal angle (HEA) -9°. **b** Medial screw hemi-epiphysiodesis changed the HEA to 5° in 1 year. **c** At age 10.9 years, the HEA remained at 25°

We could correct HEA by up to 35° even in hips with negative values of HEA preoperatively (Fig. 2). However, this does not imply that every caput valgum deformity can be corrected with screw hemi-epiphysiodesis. For the hips with an established lateral bony bridge or the hips close to skeletal maturity, screw medial hemi-epiphysiodesis would have only an effect preventing the progression of caput valgum, but not correcting the deformity. In such cases, additional procedures need to be considered for significant preexisting caput valgum.

Chang et al. reported that a bony bar formed across the epiphyseal plate along the screw tract in 5 of 8 pigs’ hips and a fibrous band formed in another three specimens [14]. In another animal study, the hip which underwent screw hemi-epiphysiodesis showed severe histological changes with epiphyseal plate closure over half the section [15]. In accordance with previous results, caput valgum did not recur in the hips that underwent screw removal before skeletal maturity even without a visible bony bar on plain radiographs. These findings suggest the permanent effect of screw hemi-epiphysiodesis, even after removal of the screw.

Screw medial hemi-epiphysiodesis in addition to preexisting lateral growth disturbance is expected to suppress the longitudinal growth of the femur. Although important determinants of skeletal growth such as gender and age were diverse, LLD which existed preoperatively decreased by 2.4 mm/year over the follow-up period, which was similar to the normal growth rate of the proximal femur [33]. Even though shortening of the leg makes the ipsilateral hip in a position of relative abduction to the pelvis which is favorable condition for the hip with DDH, the decision on screw hemi-epiphysiodesis should be made cautiously in young patients.

There were no complications associated with screw hemi-epiphysiodesis in our study. Other studies with cerebral palsy patients [18] or with DDH patients [13] also reported no complications. Since the height of the inferomedial part of the epiphysis is low due to caput valgum deformity, screw placement can sometimes be tricky. Therefore, we usually performed an intraoperative arthrogram and checked the position of the screw tip in various positions of the hip to avoid penetration of the articular cartilage.

This study had several limitations. Firstly, because it was a retrospective case series, age at screw placement and follow-up period were variable, which might lead to bias in assessing the effect of a transphyseal screw. And our cases did not compare with an untreated group of hips with caput valgum which can serve as a control group. Secondly, ~40% of patients did not reach skeletal maturity (7 of 18). Next, although radiographs were taken with the pelvis in acceptable positions and the hip in patella facing forward, the shape of the proximal femoral physis varied by femoral rotation, which may affect measurements of some of the radiologic parameters. And a femoral head which is a three-dimensional organ was evaluated two-dimensionally only using X-ray. Lastly, although it was the largest study to date investigating screw medial hemi-epiphysiodesis, the sample size was relatively small for powerful statistical analysis [13, 17, 18].

## Conclusions

Percutaneous medial hemi-epiphysiodesis using a transphyseal screw can correct caput valgum associated with DDH effectively and safely. However, this procedure does not correct coxa brevis deformity and does not appear to make a significant impact on acetabular morphology and subluxation.

## Abbreviations

AP: Anteroposterior
ATD: Articulo-trochanteric distance
CHDD: Center-head distance discrepancy
CI: Confidence interval
DDH: Developmental dysplasia of the hip
HEA: Hilgenreiner-epiphyseal angle
HSA: Head-shaft angle
LCEA: Lateral center-edge angle
LLD: Leg-length-discrepancy
MJS: Medial joint space
NSA: Neck-shaft angle
